# Variable EBV DNA Load Distributions and Heterogeneous EBV mRNA Expression Patterns in the Circulation of Solid Organ versus Stem Cell Transplant Recipients

**DOI:** 10.1155/2012/543085

**Published:** 2012-12-30

**Authors:** A. E. Greijer, S. J. Stevens, S. A. Verkuijlen, H. Juwana, S. C. Fleig, E. A. Verschuuren, B. G. Hepkema, J. J. Cornelissen, R. A. Brooimans, L. F. Verdonck, J. M. Middeldorp

**Affiliations:** ^1^Department of Pathology, VU University Medical Center, De Boelelaan 1117, 1081 HV Amsterdam, The Netherlands; ^2^Department of Clinical Genetics, Academic Hospital Maastricht, 6202 AZ Maastricht, The Netherlands; ^3^Department of Pulmonary Diseases, University Medical Centre Groningen, 9700 RB Groningen, The Netherlands; ^4^Department of Laboratory Medicine, University Medical Centre Groningen, 9700 RB Groningen, The Netherlands; ^5^Department of Hematology, University Medical Center Rotterdam, 3000 CA Rotterdam, The Netherlands; ^6^Department of Hematology, University Medical Center, 3508 GA Utrecht, The Netherlands

## Abstract

Epstein-Barr virus (EBV) driven post-transplant lymphoproliferative disease (PTLD) is a heterogeneous and potentially life-threatening condition. Early identification of aberrant EBV activity may prevent progression to B-cell lymphoma. We measured EBV DNA load and RNA profiles in plasma and cellular blood compartments of stem cell transplant (SCT; *n* = 5), solid organ transplant recipients (SOT; *n* = 15), and SOT having chronic elevated EBV-DNA load (*n* = 12). In SCT, EBV DNA was heterogeneously distributed, either in plasma or leukocytes or both. In SOT, EBV DNA load was always cell associated, predominantly in B cells, but occasionally in T cells (CD4 and CD8) or monocytes. All SCT with cell-associated EBV DNA showed BARTs and EBNA1 expression, while LMP1 and LMP2 mRNA was found in 1 and 3 cases, respectively. In SOT, expression of BARTs was detected in all leukocyte samples. LMP2 and EBNA1 mRNA was found in 5/15 and 2/15, respectively, but LMP1 mRNA in only 1, coinciding with severe PTLD and high EBV DNA. Conclusion: EBV DNA is differently distributed between white cells and plasma in SOT versus SCT. EBV RNA profiling in blood is feasible and may have added value for understanding pathogenic virus activity in patients with elevated EBV-DNA.

## 1. Introduction

Posttransplant lymphoproliferative disease (PTLD) is a severe, (pre-)malignant complication in transplant recipients, caused by Epstein-Barr-virus- (EBV-) driven B-cell proliferation in periods of defective T-cell mediated immune surveillance [1–3]. The pivotal role of EBV in PTLD pathogenesis is illustrated by the expression of EBV latent genes within PTLD lesions, including the abundant small noncoding EBER1, 2 RNA, and the essential EBNA1 protein being present in all proliferating B cells, and EBNA2 and LMP1 oncogenes being expressed in only a subset of these cells [2, 3]. At the single cell level, EBV gene expression in PTLD tissues may be heterogeneous, with coexistence of different EBV latency types as revealed by EBNA2 and LMP1 double staining immunohistochemistry [4, 5]. This heterogeneity relates to PTLD cell morphology, with relatively small PTLD cells expressing EBNA2 in the absence of LMP1, intermediate-sized cells express both EBNA2 and LMP1 (latency-III or growth program), and with larger immunoblastoid PTLD cells expressing LMP1 only in the absence of EBNA2 (i.e., default program or latency type II). The latter cells predominate and may have Hodgkin's Reed-Sternberg (HRS) cell-like morphology and a high proliferative capacity. In addition, occasionally cells show evidence of lytic viral replication [5, 6].

PTLD affects both solid organ (SOT) and stem cell (SCT) transplant recipients, but differs in pathogenesis. In EBV naïve SOT patients, the virus can be derived from the graft, blood transfusions, and external sources. However, in EBV carrying SOT recipients PTLD generally originates from reactivating endogenous virus from latently infected B cells of the host itself and frequently localises to the transplanted organ [7]. In SCT patients, EBV might be mostly derived from donor B cells or exogenous sources, since the endogenous virus can be cleared by pretreatment conditioning regimes [8]. In addition, paediatric patients developing PTLD frequently are virus negative prior to transplantation indicating EBV to originate from donor materials. In SCT EBV-driven PTLD causes a more diffuse and systemic disease initially resembling infectious mononucleosis or tonsillitis and may present at multiple locations. PTLD in SCT usually develops early after transplantation due to severe immunosuppression and delayed immune reconstitution [9]. 

Although PTLD is a highly progressive disease, it can be reverted at early stages, and preemptive treatment is indicated. This can be achieved by temporary lowering of immunosuppression, by infusion of EBV-reactive T cells or by Rituximab treatment combined with reduced immune suppression [9]. Most PTLD patients show elevated levels of EBV DNA in blood before onset of the disease. EBV DNA load monitoring enables identification of the earliest stages of EBV-driven B-cell hyperplasia [10–12]. EBV DNA is located either in circulating cells or is present as cell-free fragmented DNA in plasma. There is no evidence of EBV-DNA being associated with cell-free infectious virions in blood. Cells harbouring EBV DNA are likely to be B cells [13–15], but occasionally T cells, monocytes, and immature dendritic cells can be infected as well [16–18]. Whether circulating EBV carrying cells in PTLD patients directly reflects the tissue-bound PTLD cells in terms of viral gene expression is not well defined [19]. A role for lytic EBV replication in PTLD initiation is indicated in mouse models [20, 21], but may not be relevant in transplant settings using prophylactic antiviral agents [11, 12, 22, 23]. In fact, very little is known about gene-expression profiles in circulating EBV-positive B cells during (early stages of) PTLD and related lymphoproliferative disorders. Generally a direct concordance is assumed between tissue-associated and circulating B cells, but this remains to be established more firmly.

The aim of this study was to define the viral DNA compartmentalisation in blood cells or plasma (or both) and to describe viral gene expression patterns in circulating lymphoid cells in SCT and SOT recipients with and without PTLD. Therefore, unfractionated whole blood, plasma, and isolated B- and T-cell fractions were quantitatively analysed for EBV DNA. In addition, we determined the EBV transcriptional phenotype in blood of PTLD patients and patients with early stage B-cell hyperplasia as identified by circulating EBV DNA load elevations.

## 2. Materials and Methods

### 2.1. Clinical Specimens

Clinical samples were obtained from stem cell transplant recipients at the University Medical Center Utrecht, the Netherlands, and Erasmus Medical Center, Rotterdam, the Netherlands, and from lung transplant recipients at the University Medical Centre, Groningen, the Netherlands. Whole blood samples were collected prospectively as part of the routine diagnostic monitoring in both SOT (*n* = 15) and SCT patients (*n* = 5) using standard procedures that have been described before [22, 23]. Data from SOT patient 1 (Figure 3) were described in part previously [11]. Larger blood samples for this study were taken at the earliest time point of confirmed and sustained elevated EBV-DNA load above the clinical cutoff, indicative of early-stage PTLD [24]. PTLD was confirmed by EBER-RISH on biopsy specimens as previously described [11, 22, 23]. Additionally, in some SOT patients, whole blood samples (*n* = 12) were collected during episodes of persistent high EBV-DNA load at least 6 months after transplantation (Table 1). As control for cell fractionation and FACS-analysis, whole blood and peripheral blood mononucvlear cell (PBMC) samples from a healthy EBV carrier (*n* = 1) and from a patient with EBV-positive (Granzyme-B positive) NK/T-cell lymphoma (*n* = 1) were collected as well [3, 24].

For DNA and RNA analysis 100 *μ*L whole blood was added to 900 *μ*L NucliSens lysis buffer (BioMerieux, Boxtel, The Netherlands) directly upon collection and stored at −80°C. Plasma was separated from whole blood by centrifugation and stored at −20°C. PBMCs were isolated from whole blood by standard Lymphoprep centrifugation (Greiner Bio-one, Nuremberg, Germany). PBMCs were stored at approximately 5 × 10^6^ cells/mL in liquid nitrogen in 10% DMSO- RPMI-1640 medium (BioWhittaker, Basel, Switzerland, containing 20% FCS (Hyclone, South Logan, UT)).

### 2.2. Cell Separations Procedures

Upon thawing, viable peripheral blood mononuclear cells (PBMC) were reisolated by density gradient centrifugation over Lymphoprep (Greiner Bio-one). On average 5 × 10^6^ PBMCs were used per donor. For the isolation of the B and T cells, two different procedures were performed. In the first method PBMCs were sorted in CD20, CD4, CD8, and monocytes by FACS sorting. Monoclonal antibodies against CD20, CD4, CD8, and CD14 were conjugated to, respectively, phycoerythrin (PE), allophycocyanin (APC), Peridinin Chlorophyll-a protein (PerCp), and fluorescein isothiocyanate (FITC) and were purchased from BD biosciences (Franklin Lakes, NJ). Cells were incubated with moabs for 15 min on ice. After two washing steps with PBS, cells were resuspended and sorted by FACS-Calibur. During FACS sorting, measurements were performed to check the purity of the populations. FACS data were analyzed with DIVA software (Diva). Gating of the cells was calibrated with sorted populations prepared from frozen PBMCs of a single healthy individual during each experiment and used in all sorting experiments. The sorted cells were counted and added to 0.2 mL RNABee (AMS Biotechnology, Oxon, UK). In the second method, B-lymphocytes were isolated from PBMC by CD19 Dynabead M-450 selection (Dynal Biotech, Smestad, Norway) according to the manufacturer's protocol. From the remaining cells (designated as “non-B-cell fraction”), the T-cell and monocyte-cell populations were sorted by FACS using monoclonal antibodies against CD4, CD8, and CD14 as described above. The cell populations were counted and added to 0.2 mL RNABee.

### 2.3. EBV DNA and RNA Isolation

DNA and RNA were isolated from clinical samples collected and stored in NucliSens lysis buffer by silica-based extraction as described previously [25]. For nucleic acid isolation 100 *μ*L, whole blood material was added to 900 *μ*L Nuclisens lysis buffer. After silica-based nucleic acid extraction according to the manufacturer's instruction, DNA and RNA were eluted in 100 *μ*L. In addition, for increasing the concentration in PCR of DNA from plasma specimens, DNA was isolated by Qiamp DNA blood mini kit (Qiagen, Hilden, Germany) according to the manufacturer's instructions. RNA from cellular origin was isolated using RNABee (AMS Biotechnology, Oxon, UK) as described by the manufacturer.

### 2.4. EBV DNA Load Quantification

EBV DNA copy numbers in clinical samples were determined using standardized quantitative LightCycler-based real time PCR assays, targeting conserved 213 bp (intact virion DNA) or a 99 bp regions (detects fragmented apoptotic virion DNA as well) of the viral EBNA1 gene, as described in detail [26, 27].

### 2.5. EBV RNA Amplification

EBV-encoded transcripts for EBNA1 (QK splice variants), LMP1, LMP2a and b, BARTs, BZLF1 (ZEBRA), and the cellular low-copy U1A snRNP transcript were detected using a two-step RT-PCR assay consisting of a gene-specific multiprimed cDNA synthesis followed by PCR amplification as described previously [28]. PCR products were loaded onto a agarose gel and blotted on nylon membrane. The membrane was used for hybridization with the specific *γ*32P-ATP labelled oligonucleotide probes to determine their specificity. As a positive control, U1A snRNP RT-PCR was performed on all samples as described previously.

### 2.6. Statistical Analysis

The level of viral load in plasma was compared to whole blood. The significance of differences between categories was analyzed by means of the 2-tailed Wilcoxon signed-rank test. *P* values <0.05 were considered to be significant.

## 3. Results

### 3.1. EBV Viral DNA Load in Plasma and Whole Blood

**Figure 1:**
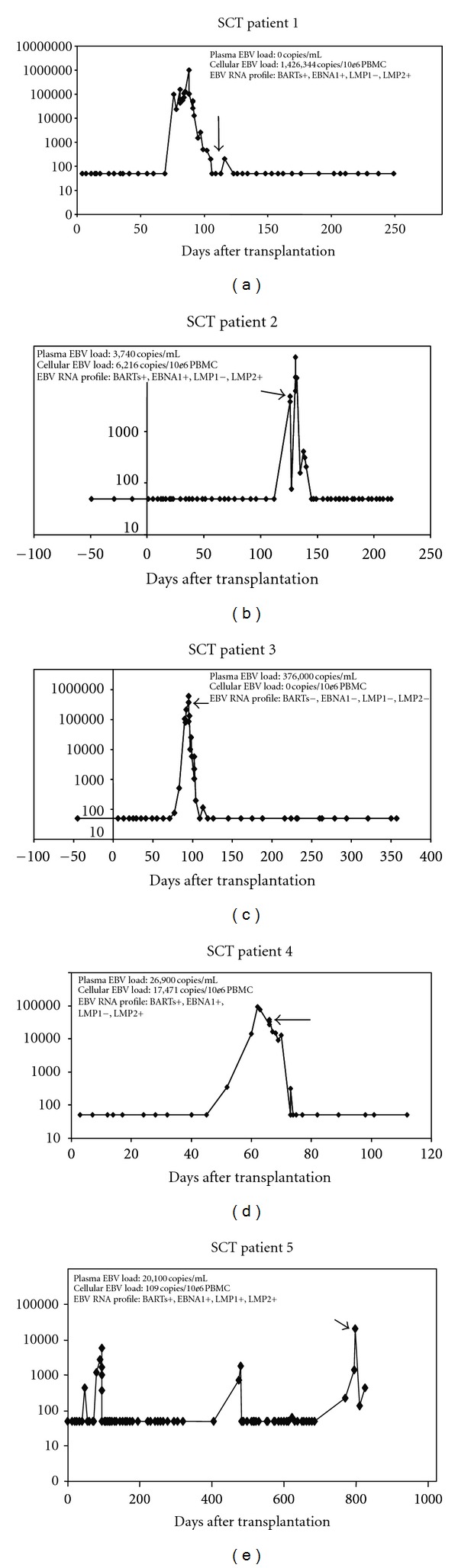
EBV RNA expression analysis in follow-up PBMC samples from SCT recipients in relation to plasma EBV DNA load kinetics. Viral DNA load in plasma was monitored by quantitative RT-PCR after stem cell transplantation (*y*-axis). At the indicated time point viral load was determined in plasma as well as in PBMCs. Presence of BARTs, EBNA1, LMP1, and 2 RNA was analysed by RT-PCR in 10e6 PBMCs.

EBV DNA loads were determined in the circulation of early onset and established PTLD patients. The DNA was measured in different compartments in the blood of SCT patients (*n* = 5) compared to SOT patients (*n* = 12). In the SCT patients, the viral DNA load was analysed weekly after stem cell transplantation in whole blood and plasma (Figure 1). EBV DNA load was determined in plasma by the 99 bp LC PCR to enable detection of fragmented DNA, whereas the 213 bp LC PCR was used for analysing intact virion DNA for cell associated EBV in whole blood. Plasma EBV DNA load was cross-sectionally compared with EBV DNA load in PBMC. Highly variable results were seen comparing PBMCs to the plasma levels of EBV DNA (Table 1). The most extremes were the total absence of EBV DNA in plasma of an SCT patient and a DNA load of 1.4 × 10^6^ copies/10^6^ cells in the simultaneously obtained PBMC sample, indicating that EBV DNA was cell associated. However, the contrary was also observed, with high levels in plasma (3.7 × 10^5^ copies/mL) and undetectable levels of EBV DNA in PBMC fraction of the same patient. No clear relation was found between EBV DNA copies in the whole blood (or PBMCs) and the plasma of the SCT patients. 

**Figure 2:**
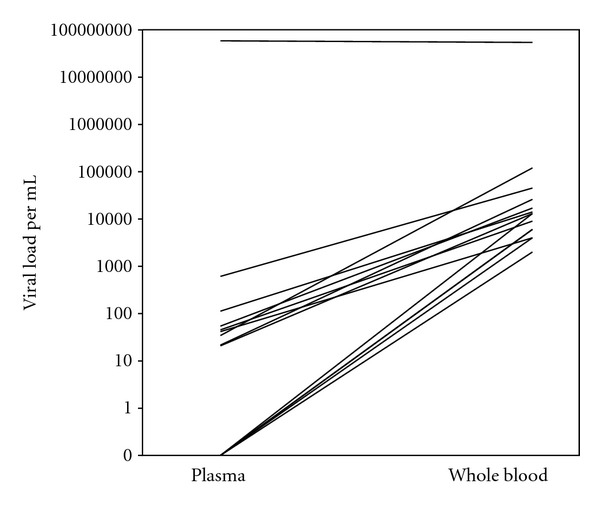
EBV DNA load analysed by quantitative PCR in whole blood compared to plasma of SOT patients (*n* = 15).

In the SOT patients, the EBV DNA load was mainly detected in whole blood (Figure 2) and only sporadically in plasma. In most cases, the whole blood contained higher levels EBV DNA copies than the parallel plasma (*P* = 0.028). This showed that the circulating EBV DNA is mostly cell associated in SOT patients.

### 3.2. EBV RNA Profiling of Circulating Cells in Transplant Patients

In SCT patients having high levels of EBV DNA in the cellular fraction (4 patients), EBNA1 and BART RNA could be detected (Figure 1). Three of the patients had LMP2 positivity, whereas LMP1 RNA was not detectable. One patient had LMP1 expression but LMP2 RNA was absent. The patient without EBV load in the cellular fraction, who was however positive for EBV DNA in the plasma (3.8 × 10^5^ copies/mL), showed no expression of EBV related RNAs in the whole blood sample.

**Figure 3:**
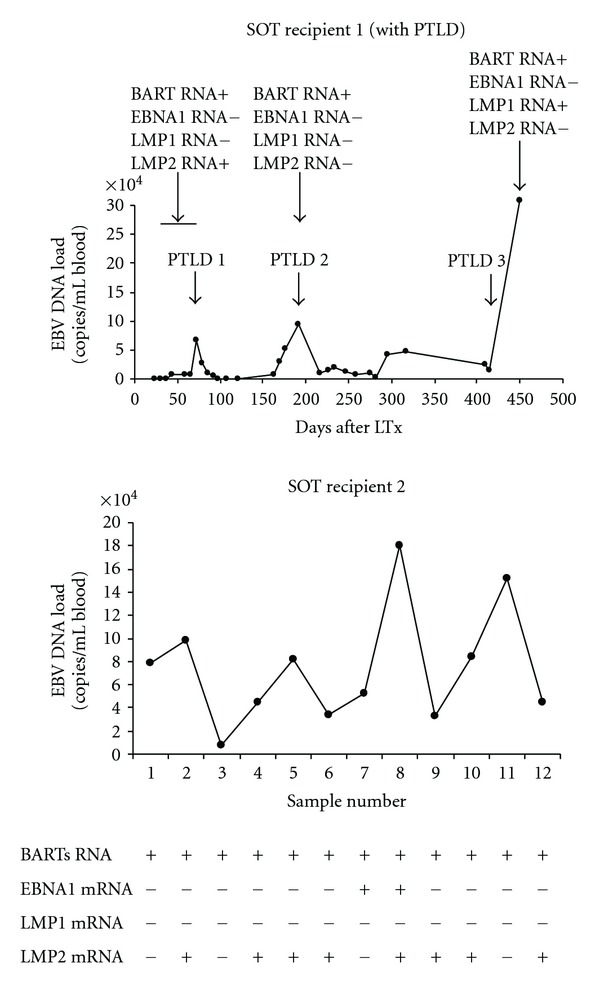
EBV RNA expression analysis in follow-up PBMC samples from two SOT recipients in relation to EBV DNA load kinetics. Viral load in whole blood was monitored by quantitative PCR after solid organ transplantation. Presence of BARTs, EBNA1, LMP1, and 2 RNA was analysed by RT-PCR in 10^6^ PBMCs at the indicated time points.

Since the elevated EBV DNA loads in SOT patients were largely associated with circulating cells, the transcriptional phenotype of EBV was analysed in the PBMCs of SOT patients. Two patients were followed in time after they received a lung transplantation. EBV DNA load and RNA analysis is shown in Figure 3. From a larger group of SOT patients (*n* = 15) with suspected PTLD early after transplantation, RNA profiles were analyzed in whole blood samples at time points of elevated (rising) EBV DNA load, prior to reducing immune suppression as a preemptive treatment. For analyzing the transcriptional activity of EBV, multiprimed RNA profiles were made as described earlier [28]. BART RNA was detected in all cases, whereas EBNA1 was only detectable in 2 subsequent samples in one patient. Transcription of the major EBV-encoded oncogene LMP1 in circulating cells was only seen in 1 patient and coincided with peak levels in EBV DNA load and coexpression with the LMP2 gene at end-stage PTLD, which was confirmed by tissue biopsy. The expression of LMP2 RNA was temporarily detected in 5 additional patients, who had a reversible PTLD, confirmed by the detection of EBER-RNA in the biopsy but disappearing after lowering immunosuppression. No ZEBRA mRNA was detected (data not shown).

### 3.3. DNA and RNA Analysis in Patients with Chronic High EBV Load after SOT

An additional group of SOT patients (*n* = 12) did not show episodes of PTLD, although the EBV DNA levels persisted at a high level (>2000 c/mL blood) for several weeks. Overall, these patients were monitored for a period of 1–14 years after transplantation. The immune suppression of all cases was preemptively lowered after detecting twice an elevated (>2000 c/mL) and increasing EBV viral load. Results are shown in Table 2. The RNA from PBMCs was positive in all cases for the low copy number cellular U1A transcript serving as an internal positive (quality) control. Among the EBV specific RNAs detected were BARTS in 10/12 of cases and LMP2 mRNA in 2/10 cases, whereas LMP1 mRNA was detected in only 1 patient during high levels of circulating EBV DNA load with EBV DNA presence in both plasma and PBMC fractions. There was no indication of circulating dividing infected cells in any of the patients, since the expression of Qp-driven EBNA1 RNA could not be detected. Viral lytic replication was absent as well since the ZEBRA mRNA was negative in all samples.

### 3.4. Origin of EBV DNA in SOT Patients with Chronic High EBV DNA Load

Chronic high levels of circulating cell-associated EBV DNA were observed in a subset of SOT patients (*n* = 8). The origin of the persistent high DNA loads was unclear and no signs of localised PTLD or progressive systemic lymphoproliferation was detected. Therefore, the origin and cellular distribution of the elevated circulating EBV DNA was analysed. Stored PBMCs were isolated and subsequently sorted into B and T cells. The cell sorting was performed in 3 patients using CD19 beads to select the B cells after which the non-B-cell fraction was sorted by FACS into CD4 and CD8 T-cells and monocytes. In the other 5 patients, PBMCs were directly sorted by FACS into CD20 (B cell), CD4-, and CD8-positive (T cell) and CD14 positive (monocyte) fractions. Gates were determined by the analysis of sorted PBMC of a healthy donor, frozen in aliquots, and thawed for each experiment. During FACS sorting, sorted populations were checked for purity by direct FACS analysis in between the procedure of sorting. The purity of the cell fractions ranged from 85% to 100%. The contamination was analyzed for markers of B and T cells and monocytes. In the fractions that were not 100% pure, the contaminating cells were not identified as B, T cells, or monocytes (all <1%). PBMCs of a patient with EBV-positive NK/T lymphoma, known to have a high viral load in T cells, were analysed as a positive control.

**Figure 4:**
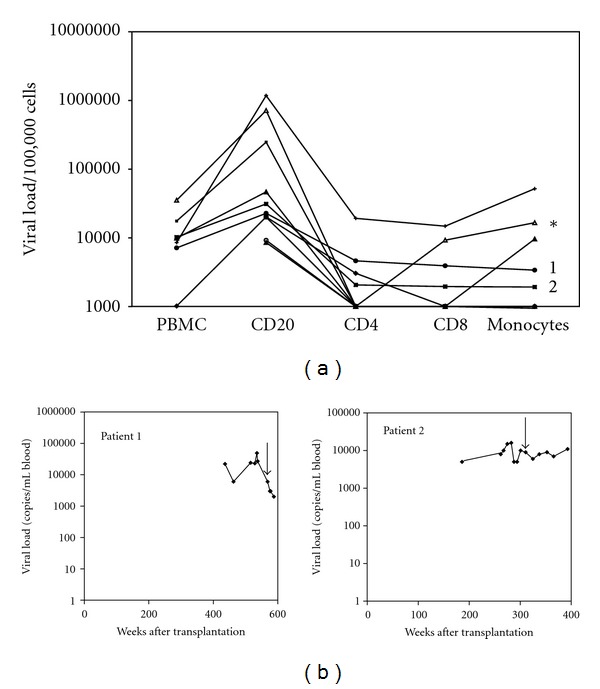
(a) Localisation of EBV DNA in PBMCs of 8 SOT patients sorted into B cells, T cells, and monocytes. As control PBMCs of a patient with NK/T lymphoma was used, indicated with an asterix. PBMCs were sorted by FACS in monocytes and B and T cells. Viral load was determined in 10e5 sorted cells. The purity of the sorted cells was higher than 85%. (b) EBV DNA load in follow-up samples of SOT patients. The arrow indicates the time point of sampling for the analysis of the viral load in the B cells, T cells, and monocytes.

The viral DNA load in the sorted cells of the patient with NK/T lymphoma was located in the B cells (1.2 × 10^4^ copies/10^5^ cells), whereas the CD8 positive T cells (1.6 × 10^4^ copies/10^5^ cells) and monocytes (2.0 × 10^4^ copies/10^5^ cells), had high levels of EBV DNA as well. Overall in this patient, monocytes, B cells, and CD8 positive T cells contained 18%, 1%, and 10% of total circulating EBV-DNA load, respectively. In the healthy donor, only occasional low level EBV DNA signals were found in the purified B-cell fraction. The EBV DNA load in PBMCs of 6 SOT patients with elevated whole blood loads was present in the B cells and EBV DNA was neither detectable in the T cells nor in the monocytes (Figure 4). In 2 patients (indicated by 1 and 2 in Figure 4), some EBV DNA could be detected in the T cells as well as in the monocytes, in addition to a high viral load in the B cells (Figure 4(a)). In these 2 patients, the overall level of viral DNA in PBMC only partly originated from B cells, since the percentage of B cells in the PBMC fraction was low (1–5%) compared to the amount of T cells (~30%) and monocytes (~20%). Although T cells harboured a lower amount of EBV DNA per cell equivalent, their contribution to the absolute viral load in whole blood may be high due to the higher numbers of T cells compared to B cells. The monitoring of the viral load in the PBMCs determined in these two patients after transplantation and the time of sampling for FACS sorting is represented in Figure 4(b). Due to limited sample sizes, no detailed RNA profiling could be done on isolated PBMC subfractions. The overall whole blood RNA patterns are shown in Table 3.

## 4. Discussion

This study shows that although both in SOT and SCT patients the development of PTLD is preceded by increases of EBV DNA load, the underlying biology of EBV-driven cell proliferation in SCT and SOT is diverse. In SOT patients the viral DNA is largely cell associated, whereas in blood of SCT patients either cell-associated or cell-free EBV DNA or (mostly) both can be detected. RNA profiles in the SCT patients were complex showing the expression of BARTs, EBNA1, and occasionally LMP1 and LMP2. Likely due to the higher immune suppression in the SCT patients, the EBV positive B cells are less silenced in their viral gene expression than in the SOT patients. Interestingly, the RNA expression profiles in circulating cells seem to show a more restricted pattern as compared with the PTLD biopsy [4–6], suggesting EBV transcriptional silencing before circulating cells leave the lymphoid tissue environment [14, 15]. The absence of EBNA1 mRNA in most whole blood or PBMC specimens indicates that the circulating cells are not replicating and are not simply reflecting the proliferating cells present in PTLD tissues. This is in line with previous observations and confirms the current prevailing model of EBV persistence, where proliferation and latent gene expression are restricted to lymphoid tissues, even in immunosuppressed individuals [14, 29, 30]. 

High and rising levels of EBV DNA are frequently detected at the onset of clinically apparent PTLD in SCT patients as well as in SOT patients [8]. However, high EBV loads alone are not informative for predicting PTLD, as most patients with chronic and stable high EBV DNA loads in this study did not develop PTLD [31]. Therefore, monitoring for dynamic changes in EBV loads is more important in identifying those at risk for developing PTLD and provides more relevant information for adapting therapy [11, 22, 23, 31–34]. Although the viral load in SOT patients is largely cell associated, as demonstrated by us and others previously, in SCT patients EBV DNA is variably distributed between cells and plasma [35]. This supports prior studies, showing that in SCT plasma levels of EBV DNA can be well used for diagnosis of EBV lymphoproliferation and PTLD risk [36]. However, in some SCT patients plasma can be virus-free with parallel high levels in PBMC, as demonstrated in Figure 1, SCT patient 1. Hence, in order to avoid false negative EBV DNA load results in high risk SCT populations, we and others have advocated the use of whole blood EBV DNA testing as the preferred standardised diagnostic approach [35, 37]. We observed no apparent correlation between the amount of EBV DNA in plasma and whole blood or PBMC, since SCT patients with the highest viral loads in plasma were not necessarily the patients with a high level in the circulating cells as was suggested earlier [34]. The origin of EBV DNA in the circulation is not clear, but it may very well be derived from apoptotic cells shed at early stage in PTLD as detected in patients with nasopharyngeal carcinoma and Hodgkin's disease [38]. On the other hand, apoptotic EBV DNA may reflect local activity of cytotoxic T cells and provide a signal of emerging immune responses. In support of this, it has been shown that EBV DNA in plasma of PTLD patients is cell-free and nonencapsidated, although nucleosomal fragmentation was not demonstrated directly [39].

Whole blood can also be used for monitoring PTLD risk in SOT patients where the EBV DNA is mainly confined to circulating cells [35, 40]. The cell-associated nature of EBV DNA in SOT was shown earlier by EBER RNA in situ staining in circulating B cells of a PTLD patient [11]. Here, we confirmed the cell association of EBV DNA in SOT, since EBV DNA levels in parallel plasma samples had values below the COV level in contrast to high viral loads in whole blood. This is in line with other independent SOT studies [40–42].

The pathogenesis of PTLD reflects a complex interaction of EBV-infected cells with the (allogenic) host environment and rejection-associated inflammatory events. Previous studies on EBV RNA expression profiling in PTLD tissue indicated a type III latency (growth program) with a combined expression of EBNA1 (reflecting proliferating cells), EBNA2 (reflecting active transformation events), and LMP1 plus LMP2 (growth activation). In contrast to PTLD tissue, EBNA1 and LMP1 mRNA is almost never detected in the circulating cells, whereas LMP2 mRNA can be found more frequently in circulating cells. Only during early stage acute primary infection such as latency type III cells has been directly demonstrated in the circulation [43]. Our data suggest that the detection of LMP1 mRNA in circulating cells can be considered a danger signal reflecting premalignant EBV-driven B-cell proliferation, which requires intervention. In contrast, LMP2 can be detected more frequently and is also described to be present in healthy individuals [14]. The percentage of LMP2 positive SOT patients is lower than described earlier [30], but this may be due to the difference in age of the patients in the two studies. The biological activity of circulating EBV positive cells in both SOT and SCT patients with elevated EBV-DNA loads seems to differ from the PTLD tissue indicating that EBV carrying B cells may undergo transcriptional silencing by methylation of EBV promoters prior to entry into the circulation. 

The RNA profiles in the circulating cells of SCT patients are more diverse compared to SOT patients. In most SCT patients, RNA encoding BARTs, EBNA1, and LMP2 was detectable, whereas in only one patient LMP1 mRNA was detected. The presence of more viral RNA populations may be due to a nonfunctioning immune system which is not fully recovered yet. Therefore, it would be interesting to analyse whether this reflected the EBV RNA expression in the bone marrow. However, due to the lack of materials in the present study, this remains as yet unresolved.

EBV is known to be B-cell associated; however, in chronic EBV disease it is described that EBV can be detected in non B cells [3] such as T/NK cells and monocytes [16–18]. In this study in 13/15 chronic EBV patients, EBV was predominantly detected in the B cells. In 2 patients, EBV DNA could also be detected in the T cells (CD4 and CD8) and monocytes. EBV infection and functional alteration of monocytes in vitro which generally do not express the EBV receptor, CD21, was shown before [44]. 

In conclusion, our data show that EBV in blood of transplant patients with (chronic) elevated EBV DNA loads is mainly confined to B cells which show limited EBV mRNA expression, supporting the notion that (pre-)malignant proliferation preferentially occurs in lymphoid tissues or in the transplanted organ. These cells do circulate, but EBV genes are probably switched off prior to entry into the blood stream. In SOT patients with chronic high EBV DNA load, of EBV, predominates in the B cells, but can occasionally persist in T cells as well as the monocytes.

## Figures and Tables

**Table 1:** SOT patients with chronic high viral load. Primary infection after transplantation is indicated.

Patient	Age	Gender	Primary disease	Time after LTx	Primary EBV infection
	Years			Years	0 = no, 1 = yes
1	57	M	Extrinsic allergic alveolitis	14	0
3	13	M	Cystic fibrosis	5	1
4	21	M	Cystic fibrosis	3	0
5	19	F	Cystic fibrosis	12	0
7	41	M	Alpha-1-AT deficiency	11	0
8	32	M	Cystic fibrosis	6	0
9	52	M	Emphysema	11	0
10	23	M	Cystic fibrosis	2	1
12	36	F	Cystic fibrosis	6	0
14	42	M	Alpha-1-AT deficiency	12	0
15	46	M	Alpha-1-AT deficiency	11	0
16	9	M	Cystic fibrosis	2	1

**Table 2:** EBV DNA load determination by LightCycler PCR in simultaneously collected plasma and PBMC samples of 5 representative SCT patients.

SCT patient	EBV DNA load in *plasma * ** **(copies/mL)	EBV DNA load in *PBMC* (copies/10^6^ cells)
1	0	1426344
2	3740	6216
3	376000	0
4	26900	17471
5	20100	109

**Table 3:** RNA profiles in circulation of SOT patients with chronic high EBV loads without PTLD. EBV DNA load and RNA profiles were analysed in PBMCs. JY and B95-8 are EBV infected B cells with latency 3, which served as positive controls for RNA profile.

Patient	EBV DNA load		RT-PCR						
	(Copies/mL plasma)	(Copies/10∗E6 PBMCs)	BARTs	EBNA1 QK	LMP1	LMP2a	LMP2b	ZEBRA	U1A snRNP
1	54	482	−	−	−	−	−	−	+
3	34	4487	+	−	−	−	−	−	+
4	0	258	+	−	−	−	−	−	+
5	0	923	+	−	−	±	−	−	+
7	0	382	+	−	−	−	−	−	+
8	45	0	+	−	−	−	−	−	+
9	112	278	+	−	−	−	−	−	+
10	610	177	+	−	+	±	−	−	+
12	0	76	+	−	−	−	−	−	+
14	21	105	+	−	−	−	−	−	+
15	21	160	+	−	−	−	−	−	+
16	41	126	−	−	−	−	−	−	+

JY RNA	na	na	+	+	+	+	+	+	+
B95-8 RNA	na	na	+	+	+	+	+	+	−
NT	na	na	−	−	−	−	−	−	−
JY DNA	na	na	−	−	−	−	−	−	−
